# Hybridization and Immunology in Animals: A Review

**DOI:** 10.1002/ece3.73500

**Published:** 2026-04-14

**Authors:** Cheyenne R. Graham, Cody W. Thompson, Delbert A. Green, Kelly A. Speer

**Affiliations:** ^1^ Department of Ecology and Evolutionary Biology University of Michigan Ann Arbor Michigan USA; ^2^ Museum of Zoology University of Michigan Ann Arbor Michigan USA; ^3^ Department of Biological Sciences Northern Arizona University Flagstaff Arizona USA; ^4^ Pathogen & Microbiome Institute Northern Arizona University Flagstaff Arizona USA

**Keywords:** hybrid vigor, hybridization, immunology, introgression, one health

## Abstract

Pathogens exert strong selective pressures on their hosts by threatening their fitness and survival, driving evolutionary innovations in many aspects of host biology. The innate immune system and adaptive immunity enable many host species to withstand such pressures; however, when pathogens exceed a host's defensive capacity, the host is forced to evolve novel adaptations to survive and reproduce. One understudied mechanism for generating this novel adaptation is hybridization, a process by which beneficial immune alleles may introgress into hybrid offspring and, via backcrossing, spread through parental populations. In this review, we synthesize evidence across diverse animal systems showcasing how hybridization shapes immunological traits. We highlight cases where hybrid offspring exhibit enhanced tolerance or resistance to pathogens relative to their parent species, and identify gaps in research needed to further examine hybridization as a pathway for species to persist in the face of infectious disease.

## Graphical abstract

Hybridization can reshape immune function by introducing novel genetic variation and combining parental immune traits. Across animal taxa, this process may influence resistance, tolerance, and pathogen dynamics, with important implications for disease ecology and One Health. This review synthesizes current evidence and highlights key gaps in understanding how hybridization affects host immunity.

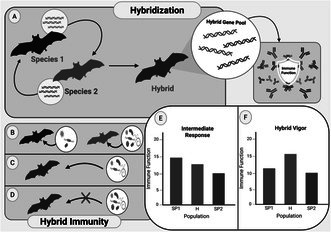

## 1 Introduction

Organisms must balance navigating external environmental pressures with defending against viruses, parasites, and bacteria (hereafter collectively referred to as pathogens unless otherwise specified), all of which directly impact host fitness (Robar et al. 2010; Gómez and Nichols 2013). Evolution has shaped both the immune system and host–pathogen interactions, with co‐evolution producing a mosaic of responses across host and pathogen species (Schmid‐Hempel 2021). As a result, host species vary in their susceptibility to different pathogens and in how they respond to infection (Haley 2003; Hawash et al. 2021). Hybridization represents a unique evolutionary process that can also influence immunological strategies and host–pathogen dynamics. Some evidence suggests that hybrids have enhanced immune responses to infection, largely because of the acquisition of advantageous immune genes from both parent species that may facilitate resistance (Wiley et al. 2009). However, hybrid immunity does not always surpass parental performance (Fritz et al. 1999). If the parental species are genetically incompatible, this can reduce overall fitness in hybrids, a phenomenon known as outbreeding depression (Šimková et al. 2015; Tolman et al. 2019). Such fitness reductions can arise from disruptions to developmental, physiological, or regulatory processes (Burke and Arnold 2001). In some cases, hybridization may also alter locally adapted immune responses through recombination, potentially reducing resistance or tolerance to specific pathogens (Porretta and Canestrelli 2023). If the hybrid's physiological or immunological phenotype is less effective at defending against pathogens than that of either parent, the hybrid phenotype may instead provide conditions more favorable for pathogen establishment, potentially leading to higher infection rates (Wiley et al. 2009). Hybridization is not limited to hosts, as parasites, including schistosomes, *Leishmania* spp. and *Fasciola*, form hybrids that can display novel, putatively adaptive traits such as higher transmissibility, broader host range and increased virulence (King et al. 2015; Lukubye and Civitello 2025) Since host hybrids often carry parasites from both parental species, they can facilitate interactions between otherwise isolated parasite lineages, influencing parasite hybridization while also showing variable infection outcomes across generations (King et al. 2015; Wolinska et al. 2008; Dedić et al. 2023). The variable impacts of hybridization on immune function and host–pathogen interactions remain poorly studied.

Although multiple reviews have addressed the ecological and evolutionary consequences of hybridization in plants, animals, and parasites (Porretta and Canestrelli 2023; King et al. 2015; Lukubye and Civitello 2025; Abbott et al. 2016; Adavoudi and Pilot 2021; Detwiler and Criscione 2010; Moulia 1999; Barton 2001; Harrison and Larson 2014; Dowling and Secor 1997; Schwenk et al. 2008; Arnold 2004), none have specifically synthesized its potential impacts on the immune systems of animals. In this review, we highlight examples from vertebrate and invertebrate systems where hybridization fostered immune adaptations, showed no measurable effect, or produced maladaptive outcomes. As research into host–pathogen relationships grows, we are gaining deeper insights into these coevolutionary dynamics and the factors promoting transmission and spillover events. This knowledge can refine One Health strategies to combat disease in human and non‐human animal populations. The link between hybridization and immunity is especially relevant in a changing world, where environmental stressors like habitat loss and climate change may alter outcomes of host–pathogen interactions. Understanding whether hybrids possess adaptive or detrimental immune traits absent in their parental species is critical, as these traits could either drive or mitigate pathogen transmission. By synthesizing findings and identifying persistent gaps, we aim to enable future research on the ways hybridization shapes host‐pathogen interactions and to explore the broader ecological and evolutionary implications of hybrid immunity.

### 1.1 Vertebrate and Invertebrate Immune System

Before examining how hybridization influences immune function, it is useful to briefly outline the key features of vertebrate and invertebrate immune systems. All animals possess innate immune defenses, which provide rapid, nonspecific protection against pathogens through epithelial barriers (Chaplin 2010), cellular responses such as phagocytosis (Buchmann 2014), and conserved signaling pathways including Toll‐like receptor signaling (Iwama and Moran 2023).

Vertebrates additionally possess an adaptive immune system characterized by antigen‐specific responses mediated by B and T lymphocytes (Cooper and Alder 2006). Adaptive immunity generates highly specific receptors through somatic recombination and supports immunological memory, enabling enhanced responses upon re‐exposure to pathogens (Chaplin 2010). Although invertebrates lack classical adaptive immunity, some taxa exhibit immune priming, suggesting that memory‐like responses are not entirely absent outside vertebrates (Kurtz et al. 2025; Melillo et al. 2018). Because immune systems are genetically complex and shaped by strong pathogen‐mediated selection (Quéméré et al. 2021; Spurgin and Richardson 2010), evolutionary processes that alter genetic composition can modify immune responses in ways that enhance, constrain, or reshape host defense strategies.

## 2 A Brief Overview of the Outcomes of Hybridization in Animals

Hybridization occurs between closely related taxa (Sequeira et al. 2020), particularly when previously isolated lineages come into secondary contact through dispersal, barrier removal, or habitat change (Grabenstein and Taylor 2018). Once thought to be rare in animals, hybridization is now recognized as a widespread and evolutionarily significant process (Adavoudi and Pilot 2021; Mitchell et al. 2019; Moran et al. 2021). This process of genetic admixture among species has been extensively examined, from classic theoretical work on hybrid zones and barriers to gene flow (Barton and Bengtsson 1986; Barton 2013) to recent syntheses addressing whether hybridization facilitates or hinders speciation (Barton 2013; Yadav et al. 2019), and it is now understood to serve as a mechanism for creating novel genetic variation (Baker and Bradley 2006).

### 2.1 Hybrid Fitness

Hybridization can increase fitness when offspring inherit advantageous alleles, a phenomenon known as heterosis or hybrid vigor (Timberlake 2013). Such benefits may include higher survival and reproduction (Johnson et al. 2010) or improved adaptation to environmental change (Kulmuni et al. 2024). In conservation, hybridization can be a source for genetic rescue, that is, small, inbred populations can gain new alleles and reduce inbreeding depression when crossed with related taxa (Zalapa et al. 2010). A classic example is the Florida panther ( *Puma concolor cougar* ), where Texas pumas (*P. c. stanleyana*) introduced into the inbred Florida population increased fertility and survival of the Florida panther, reversing inbreeding depression (Johnson et al. 2010). Such cases illustrate that although hybridization can erode distinct lineages, it can also be leveraged to conserve threatened taxa.

However, hybridization is not universally beneficial. This process can also introduce maladaptive alleles or disrupt well‐adapted genomes, underscoring its dual role as both an engine of innovation and a potential risk to population viability. Hybridization may reduce fitness when genetic incompatibilities lead to sterility or inviability. Repeated backcrossing can swamp parental gene pools, eroding unique variants and threatening species persistence (Largiadèr 2008; Meilink et al. 2015). The severity of these outcomes often depends on the relatedness of the parent species and the ecological contexts in which hybridization occurs (Whitlock et al. 2013; Pritchard et al. 2011; Chhina et al. 2022).

Hybridization may fail or produce adaptive hybrid offspring depending on the compatibility of parental alleles. Some crosses fail to produce viable offspring, whereas others yield fertile hybrids that increase effective population size or that possess greater resilience than parental phenotypes. For example, in southern Illinois, a small, isolated population of cotton mice ( *Peromyscus gossypinus* ) hybridized with white‐footed mice ( *P. leucopus* ) following habitat changes and reduced mate availability (Barko and Feldhamer 2002). The resulting hybrids increased effective population size and reproductive output, enhancing population‐level fitness and aiding recovery. Additional examples illustrating positive and negative fitness outcomes are summarized in Table 1.

**TABLE 1:** Varying outcomes of hybridization in different animal systems.

Species' Hybridizing	Outcome	Summary	Citation
**Vertebrates**			
*Lepus europaeus* (Finnish brown hare) × *L. timidus* (Mountain hare)	Positive	Finnish brown hares exhibited greater allelic variability in genomic regions associated with thermoregulation and immune function	Pohjoismäki et al. (2021)
*Crocodylus acutus* (American crocodile) × *C. moreletti* (Morelet's crocodile)	Negative	Detected hybridization between the American crocodile ( *C. acutus* ) and the endangered Morelet's crocodile ( *C. moreletii* ) using mitochondrial, microsatellite, and morphological data. However, such hybridization raised concern for *C. moreletii* , as genetic swamping could dilute or replace its unique lineage, effectively driving the species' genotype to extinction	Rodriguez et al. (2008), Cedeño‐Vázquez et al. (2008)
*Ursus maritimus* (polar bear) × *U. arctos* (brown bear)	Positive	Some hybrid offspring are fertile and could successfully produce viable second‐generation offspring. Assisting the polar bear population retains genetic diversity under changing climates	Cahill et al. (2015), Miller et al. (2024)
*Bos taurus* (Zhangmu cattle) × *B. grunniens* (yak)	Positive	Genomic analyses revealed extensive introgression from yak in the Zhangmu cattle, including alleles at key hypoxia‐response genes such as NOS2, EPAS1, and EGLN1. This introgression likely enhanced the hypoxia tolerance of Zhangmu cattle and helped maintain genetic diversity within the breed, contributing to its persistence in high‐altitude environments	Liu et al. (2021)
*Oncorhynchus mykiss* (rainbow trout) × *O. clarkii lewisi* (cutthroat trout)	Negative	Hybridization with introduced rainbow trout was associated with reduced growth and survival in native cutthroat trout, suggesting negative fitness consequences of introgression.	Muhlfeld et al. (2009)
*Gasterosteus aculeatus* (benthic) × *G. aculeatus* (limnetic): Threespine stickleback	Neutral	Wild hybrids showed no consistent reduction in growth relative to parental ecotypes, suggesting limited ecological selection against hybrids under natural conditions.	Taylor et al. (2012)
*Xenopus gilli* (Cape platanna) × *X. l. laevis* (African clawed frog)	Negative	Hybrids exhibited low fitness and rare production of viable offspring when backcrossing with either parental species.	Kobel et al. (1981)
**Invertebrates**			
*Helicoverpa armigera* (cotton bollworm) × *H. zea* (corn earworm)	Positive for the insects, negative impact on agricultural systems	Produced fertile hybrids that threaten to accelerate the spread of pesticide resistance	Gonçalves et al. (2019)
*Haliotis rubra* (blacklip abalone) × *H. laevigata* (greenlip abalone)	Positive	Hybrids exhibited faster growth and greater tolerance of fluctuating environmental conditions, advantages that emerged under variable environments rather than under stable ones	Alter et al. (2017)
*Apis mellifera* (European honeybee)	Positive	Human‐mediated admixture in European honeybees increased genetic diversity, counteracting the loss of unique variants characterizing genetic swamping	Harpur et al. (2012)

Taken together, current evidence indicates that hybridization is not a single predictable outcome but a spectrum of genetic and fitness consequences shaped by demographic history, selection, and gene flow. Recognizing this complexity is essential when examining how immune systems might evolve in hybrids, as both adaptive and maladaptive responses can emerge from the same evolutionary processes.

## 3 Hybridization as an Evolutionary Process with the Potential to Shape Host–Pathogen Interactions and Immunity

**FIGURE 1:**
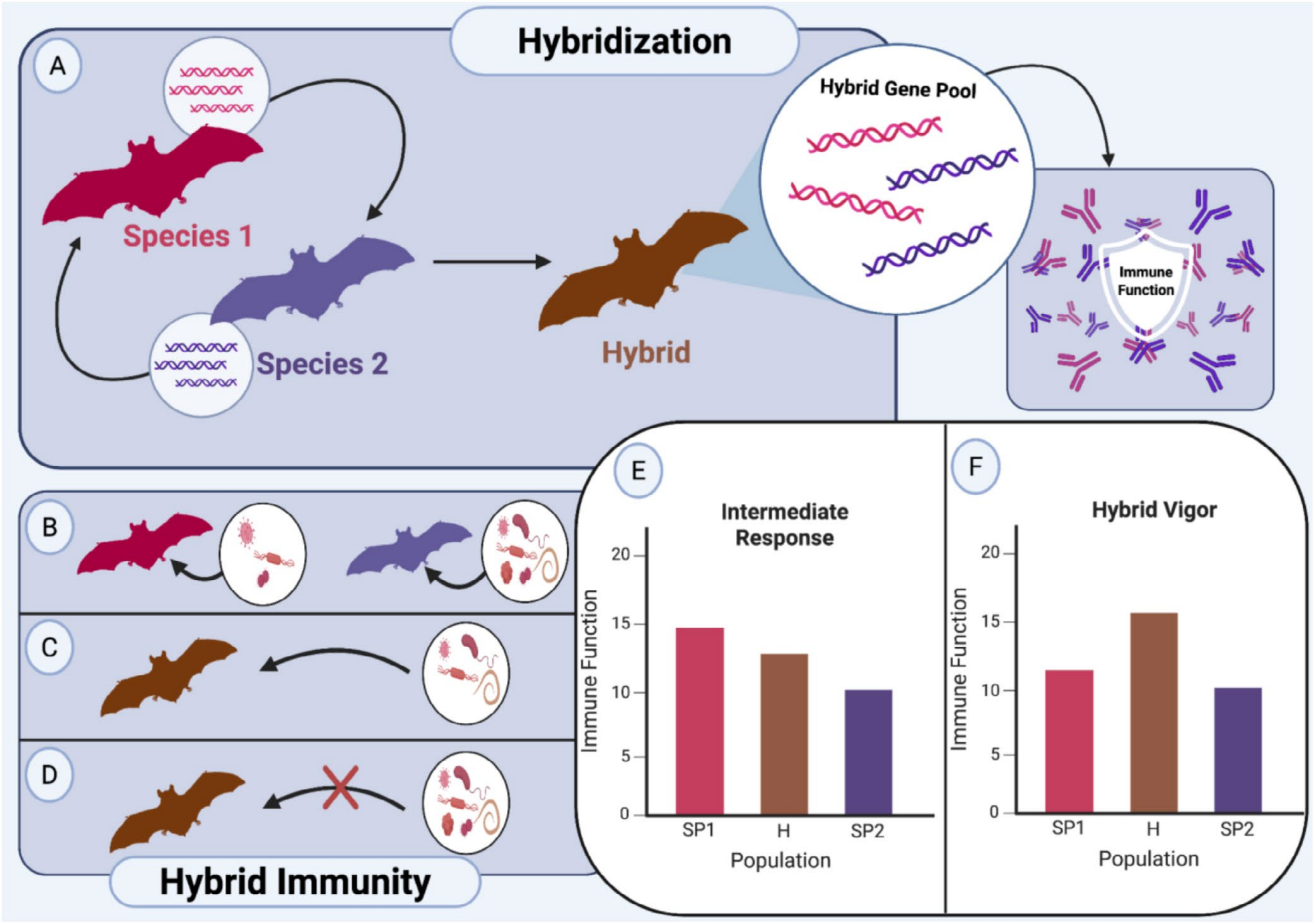
Conceptual framework for how hybridization can influence immune function. (A) Two species hybridize, combining genetic material to form a hybrid gene pool that can integrate immune alleles from both parents. (B) Each parental species shows susceptibility to its own suite of pathogens (red for Species 1; purple for Species 2). (C) Hybrids (brown) can show an intermediate immune response, with reduced infection compared to the more susceptible parent, but not full resistance. (D) Hybrids can also evolve enhanced resistance or tolerance, showing little to no infection from pathogens affecting either parent. (E) Conceptual graph illustrating the intermediate response in hybrids (H) compared with parental species (Species 1: SP1, Species 2: SP2). (F) Conceptual graph illustrating hybrid vigor, where hybrids outperform both parents in immune function under infection. (Created with Biorender).

The immune system is central to the survival of individuals and populations. When organisms encounter pathogens or environmental stressors, immune competence often determines whether they resist, tolerate, or succumb to these challenges. Outlined further below, hybridization, in the context of immunity, may combine parental alleles in ways that strengthen or weaken host defenses. Figure 1 summarizes these possibilities; hybrids may inherit immune alleles that yield stronger or intermediate responses, ranging from complete pathogen resistance to partial tolerance.

Hosts use a variety of mechanisms to counteract these threats, with the immune system serving as a primary defense against infections. However, the effectiveness of the immune system varies within individuals, between species, across space and time, and in pathogen‐specific ways (Schoenle et al. 2018; Hawley and Altizer 2011). As hosts battle pathogens, simultaneously, pathogens are battling the hosts and adapting new traits to gain an advantage and successfully bypass the hosts' defenses (Buckingham and Ashby 2022). In this cycle (i.e., the Red Queen Hypothesis or antagonistic coevolution), both host and pathogen are endlessly selected to outmaneuver the other or perish.

Hosts can adopt two main immunological strategies to counter pathogens: resistance and tolerance. Resistance involves preventing infection entirely or reducing pathogen burdens, thereby limiting the pathogen's reproductive success and therefore virulence (Råberg et al. 2009). In contrast, tolerance allows the pathogen to invade and replicate but minimizes damage to host tissue, enabling coexistence while the infection is present, whether transient or chronic (Råberg et al. 2009; Singh and Best 2021). Genetic variation and the acquisition of new genes can lead to advantageous adaptations that enable hosts to resist previously virulent pathogens or better tolerate infections (Naser‐Khdour et al. 2024; Best et al. 2008).

Novel genetic variation that encodes immune‐relevant proteins may alter the immunological strategy or success of a host in managing infection. Hybridization is one source of novel genetic variation that brings together two gene pools of distinct parental lineages, potentially introducing beneficial alleles through recombination (Twyford and Ennos 2011). These novel hybrid alleles may confer or improve resistance or tolerance in hybrid offspring relative to the parent species. Even in the context of immunity, negative consequences can arise through hybridization. This process can disrupt co‐adapted immune gene complexes or risk the loss of adaptation to local pathogen fauna (Klemme et al. 2021), thereby influencing hybrids' susceptibility and pathogen loads.

### 3.1 The Positive Effects of Hybridization on Immunity

Hybridization can lead to adaptive immunity responses in several distinct ways, including hybrid vigor, adaptive introgression, and the combination of immune traits from parental species. Tompkins et al. (2006) examined hybridization between the endangered Forbes' Parakeet ( *Cyanoramphus forbesi* ) and the more widespread Red‐crowned Parakeet ( *C. novaezelandiae* ). Using phytohaemagglutinin (PHA) skin challenges, they found that hybrids exhibited a greater relative abundance of leukocytes and a stronger response to the challenge than *C. forbesi* , though responses were comparable to those of *C. novaezelandiae* . The authors point out the conservation potential in that beneficial immune alleles from *C. novaezelandiae* may introgress into the endangered *C. forbesi* population through backcrossing, improving *C. forbesi* survival. In this way, hybridization may provide novel immune phenotypes that improve disease resistance and contribute to parental species persistence.

Hybridization can enhance resistance to infection or restore immune diversity through introgression at key loci. For instance, in a house mouse contact zone (*Mus m. musculus* × *M. m. domesticus*), parental individuals were infected with helminth parasites, but Baird et al. (2012) found that hybrid offspring carried a lower diversity and load of helminths, indicating greater resistance compared to the parental species. Similar benefits of immune‐related introgression have been documented across taxa, including introgression at MHC and other immune loci in hares, ibex, and wolves, where gene flow has restored immune diversity or introduced alleles under positive selection, contributing to immunological adaptation and population persistence (Pohjoismäki et al. 2021; Grossen et al. 2014; Münger et al. 2024). Together, these examples illustrate how hybridization and introgression can introduce adaptive immune variation that enables populations to resist pathogen pressures they might otherwise fail to withstand.

The above examples demonstrate how novel genetic variation resulting from hybridization may better enable resistance to infections. Bats have drawn significant attention for their unique immune systems among mammals and their remarkable tolerance to pathogen infections. Numerous studies have investigated the mechanisms that enable bats to harbor various pathogens, some of which are typically fatal for other mammals, while remaining relatively asymptomatic. One such study examined mouse‐eared bats (*Myotis* sp.), revealing a history of introgression within the genus (Foley et al. 2024). Notably, genes essential for immune function were found in highly introgressed regions of the *Myotis* genome. This suggests that hybridization may play a crucial role in the evolution of bats' unique pathogen tolerance. However, further research is needed to explore this relationship in greater depth.

Although hybridization may provide adaptive immune phenotypes, these benefits may diminish over generations as advantageous allele combinations are broken apart through recombination or as pathogens evolve in response. This pattern reflects segregation of alleles across generations, whereby poorly performing genetic combinations are removed by selection at the population level. Experimental studies across aquatic taxa further illustrate this dynamic: F1 hybrids in fish and mollusks displayed greater survival and immune responses to bacterial or viral challenges, whereas these benefits degraded in subsequent generations (e.g., F2 hybrids; (Zhang et al. 2019; Liang et al. 2014; Šimková et al. 2024)). Together, these examples demonstrate that hybridization can enhance immune performance in early‐generation hybrids, but that such advantages are not always stable across generations.

### 3.2 Negative and Neutral Consequences of Hybridization on Immunity

Although the previous examples highlight the potential for hybridization to strengthen immunity, its effects are not universally beneficial. In some cases, hybrids may show intermediate immune responses or no difference compared to parent species, showing that hybridization can also constrain rather than enhance immunological function. For example, Wiley et al. (2009) noted that F1 hybrids between the Pied Flycatchers ( *Ficedula hypoleuca* ) and the Collared Flycatchers ( *F. albicollis* ) did not exhibit enhanced parasite resistance. Instead, there was no significant difference between parental types and hybrids in parasite prevalence or immune response to compounds that mimic infection. The authors speculated that hybrids exhibited intermediate immune responses because the parental species possess similar immune architectures and experience comparable parasite communities. Similarly, Jones et al. (2024) examined haemosporidian infections in a contact zone between the Common Nightingale ( *Luscinia megarhynchos* ) and the Thrush Nightingale ( *L. luscinia* ). Hybrids showed intermediate infection prevalence relative to parental species; however, the small number of hybrid individuals present in this study limited statistical power. This study underscores both the possibility that hybrid immunity may be constrained rather than strengthened and the need for more robust sampling to better assess neutral outcomes.

Hybrids can exhibit opposing immunological outcomes, where enhanced resistance to one pathogen does not necessarily translate into broader immune benefits across pathogens. A clear example comes from Klemme et al. (2021), who examined hybridization between the Saimaa landlocked salmon (*Salmo salar m. sebago*) and the Atlantic salmon ( *S. salar* ). When exposed to the bacterium ( *Flavobacterium columnare* ), hybrids showed intermediate survival relative to the parental lineages, suggesting partially improved resistance. In contrast, when challenged with the trematode *Diplostomum pseudospathaceum*, hybrids exhibited higher parasite loads, indicating increased susceptibility to this parasite. The findings of Klemme et al. (2021) underscore the unpredictable nature of hybridization and its outcome for hybrid immune responses, showing that hybrids do not follow a single, consistent pattern. The literature on reduced immune performance in hybrids is limited, likely in part because inviable or sterile hybrids are transient or difficult to sample. As a result, published findings disproportionately reflect cases of hybrid vigor. This pattern could reflect a true prevalence of advantageous immune introgression, but may also be influenced by limited sampling of hybrids with reduced or intermediate immunity.

## 4 Concluding Remarks

Hybridization can generate novel immunological phenotypes, yet its consequences for immune function remain understudied. Much of the current literature centers on fish, birds, and aquatic invertebrates, whereas mammals and insects remain underexplored. In addition to addressing these taxonomic gaps, future work should assess the full range of hybrid immune outcomes, including neutral and negative effects. Further research is also needed to determine how hybridization influences pathogen–host dynamics, particularly through changes in resistance and tolerance at both individual and population levels. Expanding this research will clarify how immune systems evolve in response to pathogens and disease, with implications for pathogen transmission in wildlife and spillover to domestic animals or humans. In endangered species, such insights could inform conservation strategies if hybridization contributes to resistance or tolerance under environmental change and emerging infectious disease pressures. More broadly, understanding the immunological consequences of hybridization reframes it not just as a genetic process, but as a force shaping resilience, biodiversity, and ecosystem health.

## Author Contributions

**Cheyenne R. Graham:** conceptualization (equal), data curation (equal), formal analysis (equal), supervision (equal), validation (equal), visualization (equal). **Cody W. Thompson:** conceptualization (equal), data curation (equal). **Delbert A. Green II:** conceptualization (equal), data curation (equal), formal analysis (equal). **Kelly A. Speer:** conceptualization (equal), data curation (equal).

## Conflicts of Interest

The authors declare no conflicts of interest.

## Data Availability

Data sharing not applicable to this article as no datasets were generated or analysed during the current study.
